# Atypical Retropharyngeal Abscess of Tuberculosis: Diagnostic Reasoning, Management, and Treatment

**DOI:** 10.7759/cureus.9124

**Published:** 2020-07-11

**Authors:** Panagiota Kosmidou, Anastasia Kosmidou, Stavros Angelis, Paraskevi P Dimitriadou, Dimitrios Filippou

**Affiliations:** 1 Otolaryngology - Head and Neck Surgery, General Hospital of Thoracic Diseases, Athens, GRC; 2 Otolaryngology, Audiology–Neurotology, School of Medicine, University of Patras, Patras, GRC; 3 Pulmonology, Sotiria General Hospital of Thoracic Diseases, Athens, GRC; 4 Surgical Anatomy, National and Kapodistrian University of Athens Medical School, Athens, GRC; 5 Orthopaedics, Panagiotis and Aglaia Kyriakou Children's Hospital, Athens, GRC; 6 Orthopaedics, General Hospital Hellenic Red Cross "Korgialenio-Benakio", Athens, GRC; 7 Language, Literature and Civilization of the Black Sea Countries, Democritus University of Thrace, Komotini, GRC; 8 Surgery, National and Kapodistrian University of Athens Medical School, Athens, GRC

**Keywords:** tuberculosis, retropharyngeal abscess, aspiration, anti-tb chemotherapy

## Abstract

Retropharyngeal abscess caused by tuberculosis (TB) is an unusual life-threatening disease. We present the case of a patient, mainly complaining about dysphagia, cephalalgia, and neck pain. History, examination, and laboratory data were inconclusive. Diagnostic imaging revealed retropharyngeal abscess with atypical characteristics. Aspiration through the pharynx was performed. The aspirated fluid sent for microscopic examination by polymerase chain reaction (PCR) gene probe, revealed Mycobacterium tuberculosis (MTB). Subsequently, the patient was treated with a standard nine-month anti-TB chemotherapy. The combination of fine-needle aspiration and antitubercular agents was successful. The aim of the present study is to inform the importance of early diagnosis with targeted therapy.

## Introduction

To the best of our knowledge, a rise in the cases of extrapulmonary tuberculosis (TB) has been recently noticed in Greece, due to the increased immigration from least developed countries (LDCs) during the past few years [1]. Extrapulmonary TB represented 15% of the 7.0 million incident cases that were notified in 2018, ranging from 8% in the World Health Organization (WHO) Western Pacific Region to 24% in the Eastern Mediterranean Region [2].

*Mycobacterium tuberculosis* (MTB) is a species of pathogenic bacteria in the family *Mycobacteriaceae* and the causative agent of TB [2]. There are two types of TB: pulmonary TB and extrapulmonary TB [2]. Extrapulmonary TB is located in various organs besides the lungs [2]. TB of the retropharyngeal space is rare [3]. Retropharyngeal space located between prevertebral cervical fascia and alar fascia lies in the posterior pharyngeal wall [1]. It enhances from the skull base into the mediastinum, whereas laterally, it is defined by the carotid sheath [1].

We present the case of a patient with a tuberculous retropharyngeal abscess. Diagnostic reasoning and treatment protocol followed are analyzed.

## Case presentation

A 20-year-old Afghan origin male immigrant was referred to the Otolaryngology - Head, and Neck Surgery Department from a hosting structure in Greece, where he had been transferred for the last month. He complained of dysphagia, cephalalgia, and neck pain during the past two months. He did gargling with warm water and salt and received mild painkillers on his own initiative. No history of cough, chest pain, fever, or loss of appetite was reported. Furthermore, the patient noted no signs of trismus or difficulty in breathing. He reported no known allergies and no smoking or alcohol drinking habits. Overall, medical and family history was unremarkable.

**Figure 1:**
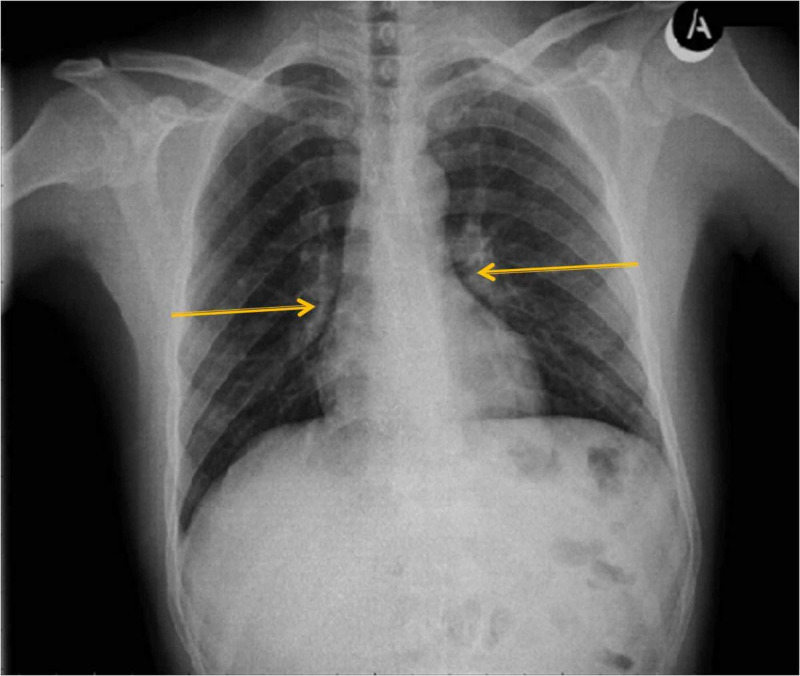
Chest X-Ray displaying nothing remarkable, except for pulmonary portal dilatation of both lungs (yellow arrows).

On arrival, vital signs were within normal range (body temperature 36.6°C, heart rate 90 beats/min, blood pressure 117/75 mmHg). Palpation of both sides of the neck revealed tenderness, though, softness and no lymph nodes were noted. Examination of the oral cavity did not reveal any pathological finds and no projection of the posterior pharyngeal wall was observed. Endoscopy (flexible nasendoscopy) of the larynx displayed normal morphology and normal functionality of vocal cords. The lungs were clear to auscultation and no neurological deficit was noted on initial clinical examination. Basic laboratory data were within normal values: hemoglobin (Hb), 14.5 g/L; hematocrit (Hct), 45.5%; white blood cell count (WBC), 5,100 cells/mm3; platelets (Plt), 284,000 cells/m3; and C-reactive protein (CRP), 0.29 mg/L. Furthermore, HIV Elisa Test was negative. Chest X-ray only revealed pulmonary portal dilatation of both lungs (Figure 1).

**Figure 2:**
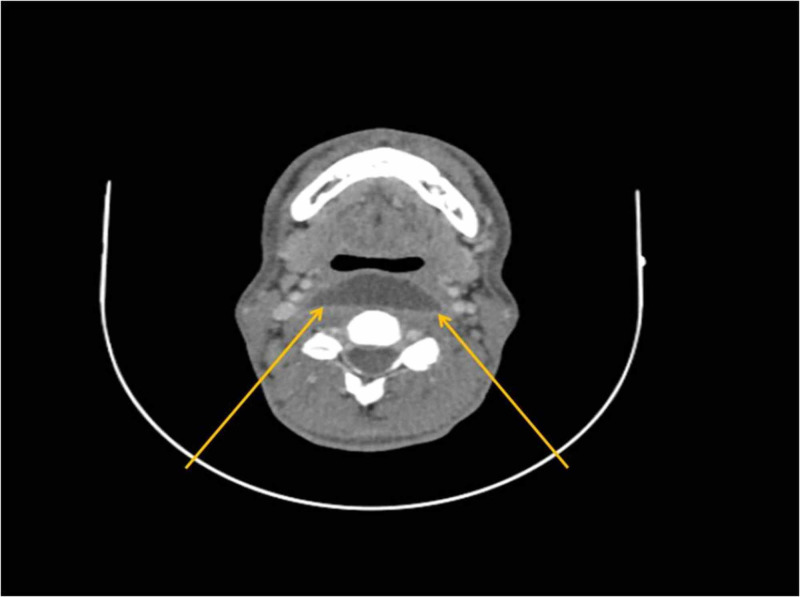
Axial CT scan of the neck, displaying an extensive abscess at the level of the second cervical vertebra in the retropharyngeal space. Yellow arrows indicate the large extent.

**Figure 3:**
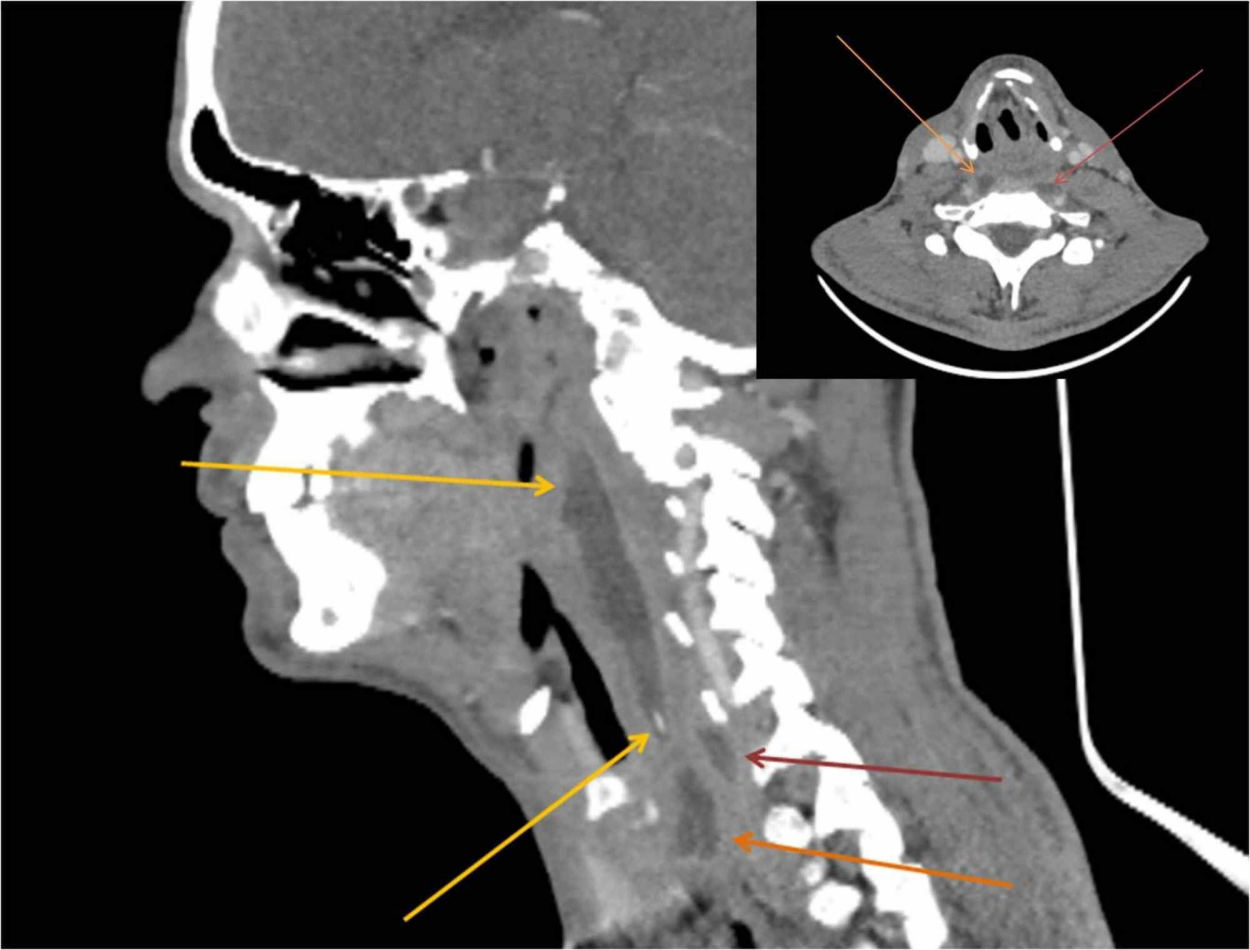
Sagittal and axial CT scan of the neck. The sagittal CT scan displays a large retropharyngeal abscess spanning from the level of the nasopharynx down to the level of the supraclavicular fossa (yellow arrows). It also expands and dives into two smaller collections in the same position (red and orange arrows). In the axial CT scan at the level of the seventh cervical vertebra, the red arrow points to an abscess located next to the seventh cervical body and the orange one points to an abscess located next to the cricoid cartilage.

**Figure 4:**
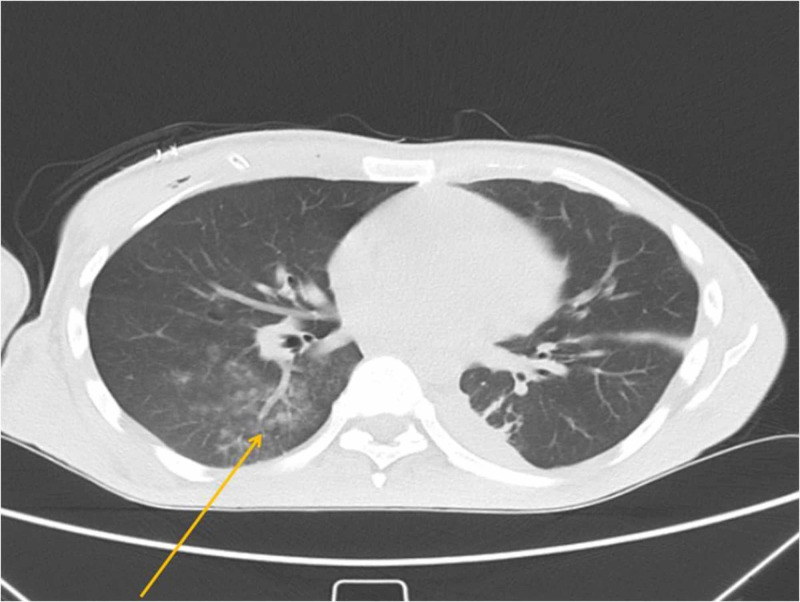
Axial CT scan of the chest revealing lesions in the form of ground-glass opacities in the upper part of the right lower lung lobe (yellow arrow).

Due to persistent dysphagia, CT of the neck and chest was performed. CT of the neck revealed an extensive multilayered retropharyngeal abscess, reaching the prevertebral space and deepening marginally into the anterior mediastinum (Figures 2-3). Chest CT demonstrated lesions in the form of ground-glass opacities in the upper part of the right lower lung lobe (Figure 4).

The patient was subjected to intraoral aspiration of the retropharyngeal abscess under local anesthesia. Sample was sent for analysis. Sixteen milliliters of pus were able to be aspirated. Immediate relief was noted as far as neck pain is concerned. Cephalalgia soon receded and the symptom of dysphagia was reported to be improved. The results of direct microscopic examination by polymerase chain reaction (PCR) gene probe demonstrated MTB.

The patient underwent antitubercular therapy. A standard nine-month course of treatment with ethambutol, rifampicin, and pyrazinamide was planned in accordance with subsequently obtained sensitivities. In summary, the patient responded to the treatment. One month later the symptoms had resolved completely. During follow-up, the patient experienced no complication and went on to make a full recovery at the end of the chemotherapy course.

## Discussion

*Mycobacterium tuberculosis* is a bacterium that causes and spreads TB [2]. It usually affects the lungs (pulmonary) or sometimes other organs (extrapulmonary) [3]. Worldwide, in 2018, approximately 7 million people were infected with TB [2]. Most patients can be cured with anti-TB treatment protocols [2].

The case of retropharyngeal infection caused by TB is rare even in the presence of extrapulmonary TB [3]. It is mentioned that only 7% of the patients with extrapulmonary ΤΒ came to affect the cervical spine [4].

The retropharyngeal space lies between the middle and deep layers of the cervical fascia and extends from the base of the skull into the mediastinum [5]. TB can be transmitted to the retropharyngeal space via the lymphatics or in rare cases via hematogenous spread [4, 6]. The latter seems to be the method of conveyance in our case, as no palpable lymph node was detected at the patient's neck.

An abscess in the neck can be fatal for the patient as it can cause airway obstruction, infection of the carotid sheath, proliferation into the mediastinum, or even septic shock [7]. The death rate of patients with these complications reaches up to 40%-50% [8]. Additional deficits common in patients with retropharyngeal TB abscess are muscle weakness and myelopathy; neither of which was encountered in our patient [3]. In this case, the patient's main concern was the symptom of dysphagia which is nonspecific and had led to a challenging differential diagnosis [9].

In conclusion, the basic steps of the diagnosis included: thorough clinical examination, imaging with CT, needle aspiration, and examination of the fluid by PCR. PCR is a validated method to confirm the presence of MTB [4]. Early diagnosis had led to a successful result with the use of antitubercular therapy and abscess drainage. 

The management of antitubercular therapy for pulmonary TB constitutes a six-month regimen of four first-line drugs: isoniazid, rifampicin, ethambutol, and pyrazinamide according to WHO guidelines [2]. For extrapulmonary TB, 9-12 months with gradual decrease in antibiotic dosage is appropriate [2].

## Conclusions

Retropharyngeal abscess by TB is rare. Unspecified symptoms and unusual location, commonly lead to delay in diagnosis and treatment. Optimal diagnosis is critical and may alter the therapeutic management. Interaction between pulmonologists and head and neck physicians is significant.
